# Increased serum NKG2D-ligands and downregulation of NKG2D in peripheral blood NK cells of patients with major burns

**DOI:** 10.18632/oncotarget.6789

**Published:** 2015-12-29

**Authors:** Josef Haik, Gil Nardini, Noga Goldman, Gilli Galore-Haskel, Moti Harats, Isaac Zilinsky, Oren Weissman, Jacob Schachter, Eyal Winkler, Gal Markel

**Affiliations:** ^1^ Department of Plastic Surgery, Tel Aviv University, Tel Aviv, Israel; ^2^ Talpiot Medical Leadership Program, Tel Aviv University, Tel Aviv, Israel; ^3^ Sackler Faculty of Medicine, Tel Aviv University, Tel Aviv, Israel; ^4^ Department of General Surgery C, Tel Aviv University, Tel Aviv, Israel; ^5^ Ella Institute of Melanoma, Sheba Medical Center, Ramat Gan, Israel

**Keywords:** burns, immune suppression, natural killer, lysis receptors, MICB, Immunology and Microbiology Section, Immune response, Immunity

## Abstract

Immune suppression following major thermal injury directly impacts the recovery potential. Limited data from past reports indicate that natural killer cells might be suppressed due to a putative soluble factor that has remained elusive up to date. Here we comparatively study cohorts of patients with Major and Non-Major Burns as well as healthy donors. MICB and ULBP1 are stress ligands of NKG2D that can be induced by heat stress. Remarkably, serum concentration levels of MICB and ULBP1 are increased by 3-fold and 20-fold, respectively, already within 24h post major thermal injury, and are maintained high for 28 days. In contrast, milder thermal injuries do not similarly enhance the serum levels of MICB and ULBP1. This kinetics coincides with a significant downregulation of NKG2D expression among peripheral blood NK cells. Downregulation of NKG2D by high concentration of soluble MICB occurs in cancer patients and during normal pregnancy due to over production by cancer cells or extravillous trophoblasts, respectively, as an active immune-evasion mechanism. In burn patients this seems an incidental outcome of extensive thermal injury, leading to reduced NKG2D expression. Enhanced susceptibility of these patients to opportunistic viral infections, particularly herpes viruses, could be explained by the reduced NKG2D expression. Further studies are warranted for translation into innovative diagnostic or therapeutic technologies.

## INTRODUCTION

Major burn injuries cause substantial clinical debilitation combined with economic impact, affecting up to 2.4 million casualties per year in the US alone [1]. One of the main complications affecting the clinical course of recovery is generalized immune suppression, which further culminates in recurrent infections and delayed healing. Immune suppression following major burns has already been described more than two decades ago and is generally observed upon sustaining thermal injury to more than 20% Total Body Surface Area (TBSA) [2]. The burn-induced immune suppression involves diminished complement [3], opsonization [4], impaired killing function of neutrophils [5] and anergy [2]. The presence of putative suppressive soluble factors was reported in the sera of major burn patients [2], as well as potential involvement of macrophages and IL-6 [6], IL-10 [7] and TGFβ [8].

Natural Killer (NK) cells belong to the innate immune branch and are capable of eliminating a broad spectrum of stressed cells, including tumors and virus infected cells [9], as well as bacterial pathogens [10]. Moreover, NK cells bridge between innate and adaptive responses by secreting cytokines such as IFNγ [11] and even by antigen presentation [12]. Recently, the importance of other innate lymphocytes, γδ T cells, has been demonstrated in wound healing [13,14] and in the immunopathology of burns [15].

In the 1980's it has been demonstrated that natural killing activity is suppressed due to major burns [16,17,18]. The decreased cytotoxicity was shown to be caused by a putative soluble factor, which has never been identified [19].

Natural Cytotoxicity Receptors (NCR) and Toll-like Receptors (TLRs) allow NK cells to participate in primary defense against pathogens and to switch on the adaptive immunity [20]. NCR include several main receptors including NKG2D [20], which is particularly interesting as some of the ligands have been identified as the stress-induced proteins MICA, MICB, ULBP1, ULBP2, ULBP3 and ULBP4 [21]. The receptor's expression is not confined to NK cells (macrophages and some T cells), and it mediates recognition of damaged cells [21], transformed cells [22] and clearance of pseudomonas [23]. The TLRs play a role in NK response against various bacteria [24, 25], and a role for TLR in bacterial clearance has also been demonstrated in mice models of thermal injuries [26, 27]. It has been reported that MICA and MICB are heat-induced [28], and that excessive soluble MICA and MICB in cancer patients (shed by the tumor cells) [29] and in pregnant women (shed by the fetal trophoblast) [30] cause downregulation of NKG2D and relative immune suppression.

We hypothesized that extensive thermal injury may result in increased systemic release of NKG2D ligands and thereby cause NKG2D downregulation and relative immune deficiency. In this case-controlled prospective pilot study we investigated the concentration of MICA, MICB, ULBP1 and ULBP2, the expression of NKG2D on peripheral blood NK cells, as well as adjunct clinical parameters, in cohorts of patients afflicted with major or non-major thermal injuries, over several time points post injury.

## RESULTS

### Clinical features

Fifteen patients were included in this study, among them five females and ten males. Five of the patients were classified as major burns, while ten patients sustained burns of a lesser magnitude and were classified as non-major burns. The Major Burn patients were 31.8 ±5.8 years old on average, and had a mean burn extent of 22.3% of TBSA. Their mean hospitalization time was 36.4 days (21-52 days) and 80% of them required surgery. On the other hand, the Non-Major Burn patients were 37.3 ±14.2 years old on average, and had a mean burn extent of 10.3% of TBSA. Their mean hospitalization time was 26.8 days, and 50% required surgery (Table 1). It should be noted that two of the Non-Major Burn patients were admitted for very long periods of times (51 and 61 days) due to local complications, as compared to the other 8 patients (10-26 days) (summarized in Table 2).

**Table 1:** General features of burn patient cohorts

	Major burns	Non-major burns	*P* value
**Mean age (years)**	31.8	37.3	Not significant
**% males**	3/5 (60%)	7/10 (70%)	Not significant
**Mean TBSA (range)**	22.6% (17-30%)	10.3% (4-15%)	0.0005
**Mean hospitalization time (days)**	36.2 (21-52)	26.8 (10-61)	Not significant
**Surgery**	4/5 (80%)	5/10 (50%)	Not significant

**Figure 1:**
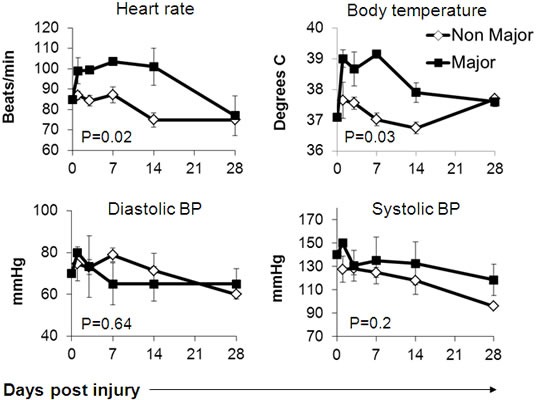
Physiological parameters in burn patients

**Figure 2:**
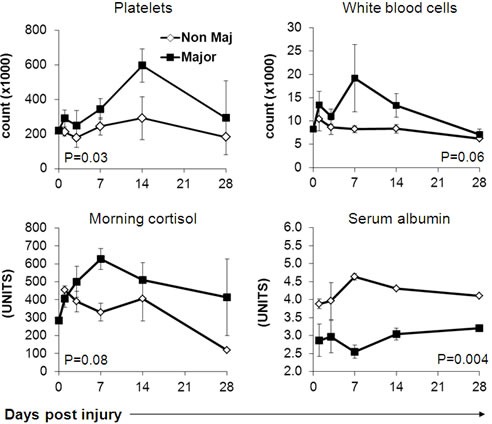
Differential basic blood tests in burn patients

**Figure 3:**
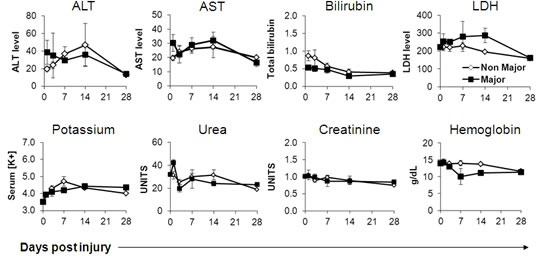
Non-differential basic blood tests in burn patients

Major Burn patients exhibited significantly higher heart rates and core body temperatures already at the first time point and onwards, with no apparent differences in systolic or diastolic blood pressure measurements (Figure 1). The severe systemic stress was also evident in laboratory studies, including significantly higher platelet and trends towards higher leukocyte counts, as well as higher blood cortisol levels in the major burn patients (Figure 2). Accordingly, serum albumin was significantly lower in these patients (Figure 2). There were no significant differences between the two groups in the level and kinetics of other parameters, including liver enzymes, serum bilirubin, LDH, blood electrolytes, renal function tests and hemoglobin (Figure 3). The vital sign records of 3 patients (A, F, M on Table 1) could not be obtained because their records have not been computerized.

**Table 2:** Patient characteristics

				Percent of burn						
Pat.	Age	Sex	Cause of burn	I	II	III	TBSA	Body area	Time in hospital	Surgery
**A**	50y	f	Scald burn	1%	2%	1%	4%	head, ear	51 days	Yes
**B**	37y	m	Open fire	1%	5%	5%	11%	leg	18 days	Yes
**C**	28y	m	Scald burn			4%	4%	arm	18 days	Yes
**D**	42y	m	Flash injury - open fire	3%	4%		7%	face, neck, arms	10 days	No
**E**	36y	m	Open fire		15%		15%	face, neck, arms	15 days	No
**F**	22y	f	Flash injury - open fire		14%		14%	face, arm	14 days	No
**G**	63y	m	Flash injury - open fire		10%	5%	15%	face, chest, abdomen, hand	61 days	Yes
**H**	51y	m	Flash injury - open fire	3%	12%		15%	face, arms, abdomen	19 days	No
**I**	22y	m	Gasoline combustion		12%		12%	face, neck, arms	26 days	No
**J**	22y	f	Scald burn		5%	1%	6%	chest	36 days	Yes
**K**	27y	f	Gasoline combustion		20%		20%	face, chest, hands, back	36 days	No
**L**	26y	m	Scald burn			20%	20%	hand, legs	21 days	Yes
**M**	31y	f	Scald burn + open fire		15%	2%	17%	face, limbs	31 days	Yes
**N**	40y	m	Gasoline combustion		8%	18%	26%	neck, chest, hands	52 days	Yes
**O**	35y	m	Scald burn		10%	20%	30%	limbs, torso, genitalia	41 days	Yes

Indicated parameters were recorded at given time points. The average results for each cohort of patients are shown. Error bars represent SE. Statistical significance with 2-tailed paired student's *t*-test is depicted.

Indicated parameters were recorded at given time points. The average results for each cohort of patients are shown. Error bars represent SE. Statistical significance with 2-tailed paired student's *t*-test is depicted.

Indicated parameters were recorded at given time points. The average results for each cohort of patients are shown. Error bars represent SE. Statistical significance with 2-tailed paired student's *t*-test is depicted.

### Serum MICB and ULBP1 concentrations are enhanced significantly in major burn patients

**Figure 4:**
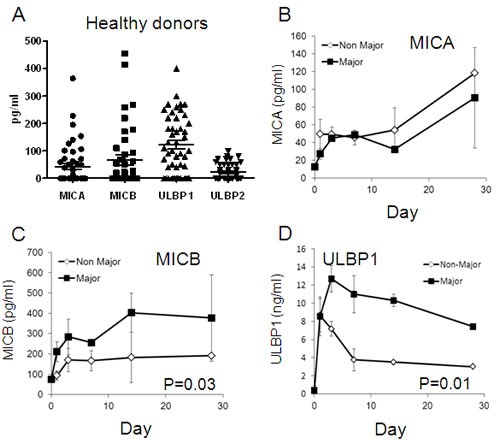
Serum NKG2D-Ligands in healthy donors and burn patients

The expression levels of serum MICA, MICB, ULBP1 and ULBP2 were studied in the peripheral blood of 50 healthy donors. The median levels of MICA, MICB, ULBP1 and ULBP2 were 40pg/ml, 80pg/ml, 130pg/ml and 10pg/ml, respectively (Figure 4A). The MICA levels of all burn patients were similar to each other and did not significantly differ from the normal values (Figure 4B). Up to a 2-fold increase was observed in day 28 in both groups, with no significant differences between them (Figure 4B). The ULBP2 levels were almost non-detectable in all burn patients (data not shown). In contrast, a rapid 3-fold increase in MICB was observed in only the Major burn patients already after 24 hours (Figure 4C). Serum MICB levels progressively increased in the Major Burn patients up to a maximum of 5-fold in day 14, and remained constant. In Non-Major Burn patients, a 1.7-fold increase was observed in day 3, which remained constant until day 28. These differences were statistically significant (Figure 4C, *p* = 0.004). For ULBP2, a dramatic elevation by more than 20-fold was observed in both groups already after 24 hours post thermal injury (Figure 4D). However, while ULBP2 serum levels were maintained at high levels in Major Burn patients up to day 28, a sharp decrease was observed in the Non-Major burn patients in the subsequent time points (Figure 4D, p-0.01).

The indicated NKG2D-Ligands serum concentrations were determined with ELISA. **A.** Concentration was determined in normal healthy donors. Each dot reflects a single individual. The horizontal line represents the median value; **B.** MICA, **C.** MICB and **D.** ULBP1 concentrations in burn patients on a time axis post burn (day 0). The average results for each cohort of patients are shown. Error bars represent SE. Statistical significance with 2-tailed paired student's *t*-test is depicted.

It should be noted that one patient (H, Table 1) in the Non-Major Burn group had exceptionally high MICA and MICB levels (10 times higher than the average). This patient also received 12 mg of Dexamethasone IV on the second day after the injury for treatment of lagyngeal angioedema. Another patient in this group (J, Table 1) had a positive blood culture for Pseudomonas Anerobius on the 7^th^ day after the burn. Positive blood cultures were obtained also for a patient in the Major Burn group (Patient O, Table 1) on days 1, 3, 7 after the burn, with antibiotic treatment initiated according to sensitivity on the 7^th^ day. One patient (N, Table 1) received 3 packed cells on the 7^th^ and 10^th^ day after the burn.

### NKG2D expression by peripheral blood NK cells is lower in major burn patients

**Figure 5:**
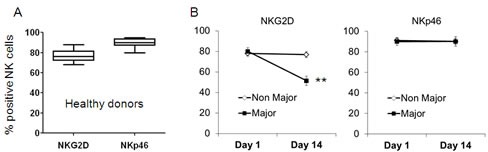
NKG2D is downregulated in peripheral blood NK cells of Major Burn patients

Next, the expression profile of NKG2D and NKp46 was analyzed in peripheral blood NK cells. Among ten healthy donors, NKG2D and NKp46 were expressed by ∼80% and ∼90% of the NK cells, respectively (Figure 5A). Remarkably, the expression of NKG2D was significantly downregulated in the Major Burn patients to ∼50% on day 14 (Figure 5B). No similar observation could be made for the Non-Major Burn patients (Figure 5B). Importantly, the expression of NKp46 remained similarly stable at day 14 as compared to day 1 in all patients (Figure 5C).

NKG2D and NKp46 expression levels on peripheral blood NK cells were determined in multi-colored FACS staining. **A.** Percentage of NKG2D or NKp46 on peripheral blood NK cells in healthy donors; **B.** Percentage of NKG2D or of **C.** NKp46 on peripheral blood NK cells in burn patients at indicated time points. The average results for each cohort of patients are shown. Error bars represent SE. Statistical significance with 2-tailed student's *t*-test is depicted.

## DISCUSSION

Immune suppression following major thermal injury is a common problem with substantial clinical impact. Most of the proposed mechanisms up to date involve diminished innate immune components, such as complement [3], opsonization [4] and neutrophil function, attributed mainly to cytokines such as IL-6 [6], IL-10 [7] and TGFβ [8].

We hypothesized that the immune suppression might be linked also directly to the mechanism of thermal injury. The expression of stress-induced ligands MICA and MICB was previously reported to be upregulated following thermal cell damage [28]. Following this rationale, we compared serum MICA, MICB, ULBP1 and ULBP2 levels, as well as NKG2D expression on peripheral blood lymphocytes in two cohorts of patients: Major burn patients ( > 15% TBSA) and Non-Major Burn patients ( < 15% TBSA). We observed the expected, significant differences in some physiological parameters, such as heart rate, between these groups (Figures 1-2). These differences are mostly secondary to the physiological response to the extensive trauma. Upregulation of serum MICB and ULBP1 concentration was documented in both groups but the kinetics was significantly different. In Major Burn patients, increasing serum MICB was evident earlier; it continued to develop longer and reached significantly higher maximal concentration (Figure 4). For ULBP1, the initial response was similar between the patient groups, but the exceptionally high concentrations were maintained only in the Major Burns group, while a sharp decrease was observed in the Non-Major Burns group. These characteristics are indicative of a “dose dependent” response and strengthen the link between the inflicted burn and increased serum MICB and ULBP1. It could be due to expression and shedding from damaged tissues or from other tissues responding to the general stress. It was published recently that thermal stress can lead to the secretion of exosomes containing NKG2D-ligands from cell lines [31]. The exact mechanism *in vivo* remains to be delineated in future studies. While moderate, late, increase in MICA was observed, there were no significant differences between the groups, suggesting an indirect effect (Figure 4). ULBP2 was entirely undetected, suggesting that it is not affected by this type of stress. To the best of our knowledge this is the first description of kinetics of these biomarkers *in vivo* in humans. It should be emphasized that as opposed to the other clinical conditions associated with high serum MICs [29, 30], the onset of pathology in burn patients is acute and definitive.

It was shown inadvertently that high serum NKG2D-Ligands can cause downregulation of NKG2D in cancer patients [29] and during pregnancy [30] by ligand-induced endocytosis [29, 30]. This culminates in relative immune suppression, which is further exploited by the cancer cells and the extravillous trophoblast of the fetus [29, 30]. NKG2D plays a major role in mediating immune response against viruses such as CMV [32], EBV [33] and HSV [34]. Although extracellular bacterial infections are the most common infectious cause of death in burn patients, viral infections, especially CMV and HSV, pose a common threat [35]. Indeed, CMV seroconversion, was reported years ago to be associated with significantly longer hospital admission and requirement for blood transfusions [36]. Further, it was recently shown in a prospective study that CMV reactivation occurs in 70% of seropositive patients with major burns [37]. There are similar associations with HSV infections [38, 39]. In addition, CMV can be transmitted *via* cadaveric skin grafts [40]. It was shown in animal models that thermally-injured animals are more susceptible to this transmission [41]. NKG2D downregulation as shown in our report might account for this sensitivity to viral infections in Major Burn patients. The peak of viral infections is between the first and third week [39], which coincides with maximal serum MICB and ULBP1 concentrations (Figure 4), and with NKG2D downregulation (Figure 5).

In conclusion, we point on a new potential mechanism of immune suppression in Major Burn patients, which could explain their susceptibility to viral infections. These findings should be corroborated in larger trials. Development of novel technologies that prevent NKG2D downregulation or enable regeneration of its expression might be beneficial in patients with major burns.

## PATIENTS AND METHODS

### Ethics

This study was approved by an Institutional Review Board of Sheba Medical Center (study number 4674) and all human subjects gave their written informed consent as required by the Declaration of Helsinki.

### Competing interests

The authors have declared that no competing interests exist. The funders had no role in study design, data collection and analysis, decision to publish, or preparation of the manuscript.

### Patients

Over a period of 3.5 years, from June 2007 until December 2011, a total of 15 patients met the inclusion criteria and were enrolled to the study. The inclusion criteria included age 18-70, thermal burn injury and arrival to the department within the first 24 hours of injury. The exclusion criteria included any condition that prevents the patient from providing an informed consent (e.g. unconsciousness, major psychiatric disorder) and chronic diseases or treatments that may affect the immune system (e.g. malignancy, type II diabetes, immunosuppressive treatment). The patients were classified into two groups of “Major” burns or “Non-Major” burns. Major burns were defined third degree burns involving more than 10% of TBSA or second degree burns involving more than 15 % of TBSA. Blood samples were collected within 24h of the injury and on days 3, 7, 14 and 28 after the burn, provided the patient was still hospitalized. The patients' vital signs and laboratory investigations were kept *via* the medical computerized records. Healthy donors served as negative controls for serum studies.

### Blood samples

All blood samples were obtained in Chemistry tubes to allow clotting, centrifuged and sera (supernatant above the gel) were collected. All serum samples were frozen in −80 Celsius until tested. Blood was analyzed for CBC, blood chemistry, morning cortisol, microbiological cultures and for concentrations of MICA and MICB. Blood samples for lymphocyte analysis were obtained in heparinized tubes (Becton Dickinson). Peripheral blood mononuclear cells were purified by standard ficol gradient centrifugation and deep frozen.

### ELISA for MICA, MICB, ULBP1 and ULBP2

Soluble MICA, MICB, ULBP1 and ULBP2 were quantified in serum samples by using commercial sandwich ELISA kits according to manufacturer's instructions (R&D Systems). Briefly, each serum sample was tested in triplicates in 96well immunoplates (Nunc), 100microliter serum in each well. The following specific antibody concentrations were used for quantification of MICA, MICB, ULBP1 and ULBP2: Capture antibody 2μg/ml, 4μg/ml, 4μg/ml, 4μg/ml, respectively; Detection antibody 0.4μg/ml, 0.4μg/ml, 4μg/ml, 2μg/ml, respectively. PBS with 1%BSA used for blocking and diluting of all antibodies.

### Flow cytometry

For staining of peripheral blood mononuclear cells with purified antibodies, 100,000 cells were incubated with 0.2μg of antibody diluted in PBS/0.5% BSA/0.02% NaN3 (FACS medium) for 1h on ice as previously described [42, 43]. Cells were centrifuged 400g/5min and supernatant was removed. The cells were incubated 30mins on ice with secondary antibodies, washed with FACS medium and analyzed with FACS calibur instrument (BD, Franklin Lakes, NJ, USA) and FlowJo or CellQuest software. The following antibodies were used: PE-anti-CD56 (DAKO, Glostrup, Denmark, clone MOC-1), FITC-anti CD3 (DAKO, clone UCHT1), APC-anti-NKG2D (eBioscience, San Diego, USA, clone CX5), APC-anti-NKp46 (eBioscience). Isotype control IgG1-FITC (DAKO), IgG1-PE (DAKO) and IgG1-APC (eBioscience) served as negative controls. NK cells were gated based on positive CD56 expression and lack of CD3 expression. At least 2,500 gated NK cells were analyzed in each reading.

### Statistics

Statistical analysis of the general properties of major and non-major burn patients, as well as of NK cell staining for NKG2D and NKp46 was performed with Mann-Whitney test. Statistical analysis of the kinetics of various physiological, routine lab or the experimental biomarkers was performed with pair *t* test. P value of 0.05 was set as significant.
